# Clinical severity of *Mycoplasma pneumoniae *(MP) infection is associated with bacterial load in oropharyngeal secretions but not with MP genotype

**DOI:** 10.1186/1471-2334-10-39

**Published:** 2010-02-25

**Authors:** Anna C Nilsson, Per Björkman, Christina Welinder-Olsson, Anders Widell, Kenneth Persson

**Affiliations:** 1 Department of Clinical Sciences, Malmö Infectious Disease Research Unit, Lund University, 205 02 Malmö, Sweden; 2 Department of Clinical Microbiology, Sahlgrenska University Hospital, 413 45 Göteborg, Sweden; 3 Malmö Department of Clinical Microbiology, Lund University, 205 02 Malmö, Sweden

## Abstract

**Background:**

Disease severity in *Mycoplasma pneumoniae *(MP) infection could potentially be related to bacterial factors such as MP genotype (MP1 or MP2; distinguished by different adhesions proteins) or bacterial load in airway secretions. We have compared these parameters in patients who were hospitalized for MP pneumonia, with outpatients with mild MP disease.

**Methods:**

MP bacterial load was measured by real-time PCR in 45 in- and outpatients ("clinical study group") in whom MP DNA had been detected in oropharyngeal secretions by PCR. In addition, genotype and phylogenetic relationships were determined. The phylogenetical assessment was done by partial DNA sequencing of the P1 gene on isolates from 33 patients in the clinical study-group where sufficient DNA was available. The assessment was further extended to isolates from 13 MP-positive family members and 37 unselected MP positive patients from the two subsequent years and two different geographical locations. In total 83 strains were molecular characterized.

**Results:**

Mean MP loads were significantly higher in 24 hospitalized patients than in 21 outpatients (1600 vs. 170 genomic equivalents/μL, p = 0.009). This difference remained significant after adjustment for age and days between disease onset and sampling. Hospitalized patients also had higher C-reactive protein levels. Mean levels were 188 vs 20 mg/L (p = 0,001). The genotype assessment showed MP genotype 1 in 17 of the 33 sequenced strains from the clinical study-group, and type 2 in 16 of these patients. Within each genotype, sequence differences were minimal. No association between disease severity and MP genotype was observed. In the extended genotype assessment, MP1 was found in similar proportions. In family contacts it was found in 53% and among patients from the two subsequent years 53% and 40%.

**Conclusions:**

A higher MP bacterial load in throat secretions at diagnosis was associated with more advanced respiratory disease in patients, but MP genotype did not influence disease severity. Both MP genotypes co-circulated during recent outbreaks in Sweden.

## Background

*Mycoplasma pneumoniae *(MP) is a major respiratory pathogen that can cause clinical disease ranging from mild upper respiratory tract infection (URTI) to severe, occasionally fatal pneumonia. MP infection may also lead to several extra-pulmonary conditions, such as myocarditis, meningoencephalitis and hemolytic anemia [1,2]. Previously, the only available method for diagnosing MP infection in clinical practice was serology, permitting a diagnosis no earlier than one to two weeks after disease onset when antibodies have developed. We have recently presented data showing that nucleic acid amplification tests (NAATs) on throat secretions have superior sensitivity to serology during the early phase of MP disease [3]. In addition, we found that the average MP load continuously declined after disease onset. Eventually all patients became negative (in their throat-samples) for MP DNA. Half of the patients had become negative after 54 days; however, one patient carried MP for 7 months.

Infectious disease manifestations may be explained by both host- and pathogen-related factors. For MP, correlates of disease severity are incompletely known. An association between the load of MP DNA and clinical severity was demonstrated in one report of ten patients, showing a higher level of bacterial genome equivalents in cases with a more severe clinical course [4] however, the results were not adjusted for age and interval between disease onset and sampling.

MP can be categorized into two genotypes, MP1 and MP2, based on the DNA sequence of the P1 adhesion protein, which is located in the cell membrane and is of vital importance for bacterial adhesion to epithelial cells [5-10]. Previous studies have suggested that these two genotypes may co-circulate during an MP outbreak [11]. Whether the clinical manifestations differ for the two MP genotypes is not known.

This study aims to determine whether MP bacterial load and genotype are associated with disease severity, to characterize oropharyngeal isolates of MP obtained during an outbreak in 2005-2006 in an urban area of Southern Sweden, and to compare these results with clinical data. In addition, the MP genotype distribution during this and other recent outbreaks in Southern Sweden was investigated and strain differences assessed phylogenetically.

## Methods

### Study population

#### 2005-2006 outbreak patients (clinical study group)

All 45 MP PCR positive individuals identified in a previous study, which compared serology and MP PCR in oropharyngeal secretions for the early diagnosis of MP infection [3], were included. These patients, consisting of 24 hospitalized patients and 21 outpatients, are henceforth referred to as the "clinical study group". These patients, all with respiratory tract infection suggesting MP infection were recruited from the Department of Infectious Diseases at Malmö University Hospital (serving Malmö city with suburban areas in southern Sweden with 360 000 inhabitants) and from four primary health care centres in Malmö city during the winter season 2005-2006 (September 20 - March 15; when rates of detected MP infections were high).

All 45 patients had their initial PCR test confirmed at least once by quantitative real-time PCR (qPCR). They underwent consecutive PCR testing on serial samples until 2 consecutive PCR negative samples were obtained. Clinical data were obtained at the first visit to the health care provider when MP infection was suspected. In some cases patients had already been unsuccessfully treated with betalactam antibiotics at that time. Information was regularly collected during the course of the disease and after resolution of symptoms, using medical records, interviews and questionnaires [3].

Levels of C-reactive protein (CRP) were analysed at first visit in 37 of the patients (20 inpatients and 17 outpatients). All CRP-results were obtained by analysing plasma samples on a Beckham Synchron LX20 (Beckham Coulter, Inc. Fullerton, CA, USA) at the Laboratory of Clinical Chemistry at Malmö University Hospital.

#### Family contacts

Household members of ten MP patients in the study were offered MP testing to estimate MP transmission. Eighteen of 22 subjects tested were MP PCR positive, all with ongoing or recent symptoms of URTI. At least one MP positive family member was found in all households screened.

None of the family contacts had sought health-care on their own initiative. They underwent no physical examination nor had their CRP levels determined but were followed by repeated PCR testing and self reported symptoms in the same manner as the patients in the clinical study group. During follow-up, all of the family contacts became PCR negative. Strains from the family contacts were only included in the assessment of genotype distribution and genetic conformity, not in the MP load evaluation.

#### MP strains collected during other seasons

For epidemiological strain comparison, MP isolates from patients from two other seasons and locations were also DNA sequenced and genotyped. These included strains from 20 unselected patients diagnosed with MP infection by PCR in Malmö during the winter season of 2007-2008 and 17 MP isolates obtained from patients during the latter half of the outbreak 2006-2007 in Göteborg, (250 km north of Malmö). These strains were only included in the assessment of genotype distribution and genetic conformity, not in the MP load evaluation.

### Sampling procedure

A cotton-tipped swab was used to obtain throat samples from the posterior wall in the oropharynx. These samples were transferred to tubes containing 1 mL phosphate buffered saline.

Written informed consent was obtained from all participants, and the study had been approved by the Lund University ethical committee.

### Laboratory methods

#### DNA extraction

DNA was extracted from the samples by the MagNa Pure LC Instrument using the MagNa Pure LC DNA Isolation kit I (Roche Diagnostics GmbH, Mannheim, Germany). Nucleic acid from 200 μL of the sample was finally obtained in 75 μL elution buffer.

#### Quantitative PCR

A quantitative real-time Taqman PCR method was applied as described elsewhere [3] with primers 435 (5'-GGCAGTCAACAAACCACGTATG-3') and 479 (5'-GGTGGTTGATGCGGTCAAA-3') together with the probe (FAM-5'-TCGCGTTCCTTTCTCCCGACGTTTT-3'-TAMRA). As a standard for quantification the PCR amplification fragment using the primers above was cloned into a plasmid using the pUC system followed by transfection of a carrier *E. coli*. The plasmid with its cloned fragment was then purified using the QIAprep Miniprep kit (Qiagen, Hilden, Germany), quantified spectrophotometrically and then included in tenfold serial dilutions as a reference curve for quantitative measurements of MP in clinical samples.

#### DNA sequencing

Thirty-three (17 inpatients and 16 outpatients) of the 45 MP positive samples from Malmö patients and 13 of the 18 family members were typed by sequencing a fragment of the gene coding for the P1 adhesion protein as described by Kong et al. [10], after being amplified with nested PCR. Sequencing could not be done in the remaining samples due to lack of DNA material. For the outer PCR the primers were P1-178 (5'-CAATGCCATCAACCCGCG-3') and MPAW1 (5'-GCGCGCATAAGGCGCATC-3'). The inner PCR utilized primers MPSW2 (5'-GTGAAACGAGGTCAAAAA-3') and MPAW (5'-ATAAGGCGCATCGTACAG-3'). The latter pair was also used as sequencing primers. The amplification products for MP1 were 606 bp and for MP2 621 bp. They were purified and sequenced using an ABI cycle sequencing system (BigDye, ABI).

### Phylogenetic analysis

Complementary sequences for each isolate, with primer sequences removed, were edited by the ABI softwares Factura and Sequence Navigator and transferred in FASTA format to BioEdit Sequence Alignment Editor (version 7.01, Tom Hall, Dept of Microbiology, North Carolina University, USA). Alignment was conducted with Clustal via the putative translated amino acids, and then translated back to DNA sequences to maintain codon integrity. Alignment induced gaps were subsequently handled as pair-wise deletions in the phylogenetic and molecular evolutionary analyses using MEGA version 3.1. Phylogenetic trees were constructed using the Neighbour-Joining method. The substitution algorithm was based on p-distances calculated by the Kimura 2-parameter method. Reference sequences were from prototype genotype 1, -M 129 - (GenBank; accession number U00089) and type 2, -TW 7-5/FH [12].

### Statistical analysis

Patients of the 2005-2006 season where divided into two groups; those hospitalized (with more severe illness) and those treated as out-patients (milder illness) and statistical evaluations of MP load in baseline samples and genotype distribution were based on this classification.

Differences in MP load and mean values of CRP were assessed by Student's t-test and the non-parametric Mann-Whitney U-test. Logistic regression was used to adjust for age and interval between disease onset and sampling. Differences in proportions of genotype were analysed by the Chi-square test.

The Statistica package (StatSoft, Tulsa, OK, USA) and the R-package (Vienna, Austria) were used.

## Results

### Patient characteristics

Characteristics of the 45 patients from the 2005-2006 outbreak are presented in Table 1. Twenty-four patients had been admitted to the hospital as inpatients and 21 were treated as outpatients. Inpatients were admitted for a median of 7 days (range: 1-21). Their median age was 37 years (range: 2-79), compared to 33 years for outpatients (range: 9-55). Among the hospitalized patients, 15/24 had peripheral oxygen saturation below 90%, and were thus given oxygen therapy. However, no patient required ventilatory support or intensive care.

**Table 1:** Characteristics of patients and family contacts.

	*Inpatients* *N = 24*	*Outpatients* *N = 21*	*Total patients* *N = 45*	*Family contacts* *N = 18*
Median age (years; range)	37 (2-79)	33 (3-66)	41 (2-79)	13 (2-65)

Underlying chronic diseases	5^a^	1^b^	6	0

Mean level of C-reactive protein (mg/L; range)	188^c^ (9-353)	20^d^ (<0,2-174)	62 (<0,2-353)	

Interval between disease onset and sampling (days; range)	10 (3-33)	17 (1-95)	13 (1-95	

Prior therapy with betalactam antibiotics	24	8	32	0

Current smoking	1	0	1	3

^a^2-Crohn's disease, 1-Parkinson's disease, 1-atrial fibrillation, 1-type 2 diabetes mellitus

^b^1-bronchial asthma

^c ^vs ^d ^- Mann-Whitney U-test - p < 0,001 and Student's t-test - p < 0,003

Hospitalized patients were slightly older than outpatients, and the proportion of women was higher (75% vs. 54%). Six patients had underlying chronic diseases (5 inpatients and 1 outpatient), but no patient had significant immunosuppression. Median number of days between disease onset and initial sampling for MP was 13 days (range: 1-95). For inpatients median number of days was 10 (range 3-33) and for outpatients 17 (range 1-95).

According to the study design, MP sampling was done when a clinical suspicion of MP infection arose, often after clinical failure of betalactam antibiotics for the treatment of community-acquired pneumonia.

All 24 hospitalized patients had received betalactam antibiotic therapy prior to MP diagnosis, compared to 8/21 outpatients. A greater proportion of hospitalized patients (23/24 vs 2/21) had undergone chest X-ray, which showed pulmonary infiltrates compatible with pneumonia in 23 cases; however, such infiltrates were also observed in the two outpatients who underwent chest X-ray. Current smoking was self-reported for 1/24 hospitalized patient, 0/21 outpatients and 3/18 family contacts.

Mean baseline CRP levels were significantly higher in inpatients than outpatients, 188 vs. 20 mg/L (p < 0.001) using Mann-Whitney U-test; p < 0.003 using Student's t-test. (Table 1).

### Disease severity

#### Bacterial load and disease severity

Among the 24 inpatients the geometric mean number of genomic equivalents in the diagnostic MP sample was 1600 (range: 14-570 000) DNA equivalents/μL throat secretion compared to 170 (range 7 - 5 400) DNA equivalents/μL throat secretion among the 21 outpatients (p = 0,009 using the Student's t-test and p = 0.039 using the Mann-Whitney U test). Differences in bacterial load at diagnosis remained statistically significant after adjustment for age and time between onset of symptom and sampling in a logistic regression model. (Table 2).

**Table 2:** Logistic regression analysis of MP DNA load at diagnosis.

	*Crude OR (95%-CI)*	*Adjusted*^a ^*OR (95%-CI)*	*P values (crude/adjusted)*
*Patients*	2.12 (1.14-3.92)	2.36 (1.08-5.18)	0.017/0.03
*Patients+ family members*	2.42 (1.16-5.08)	2.56 (1.20-5.43)	0.023/0.015

In-patients vs out-patients.

^a^Adjusted for age and time period between disease onset and first sample.

#### Genotype and disease severity

Genotype MP1 or MP2 was determined for strains from 33 patients where MP-DNA was available. Chromatograms did not indicate any mixed MP infections. The proportion of patients infected with MP1 was slightly higher among those with more severe illness, 58% (11/19) compared to patients with milder disease, 43% (6/14) but the difference was not statistically significant (χ^2 ^= 0.25, p = 0.62).

#### Genotype and bacterial load

Geometric mean number of genomic equivalents was 560 DNA equivalents/μL for MP1 and 400 DNA equivalents/μL for MP2. The difference was not statistically significant (p = 0,67).

#### Genotype distribution

**Figure 1:**
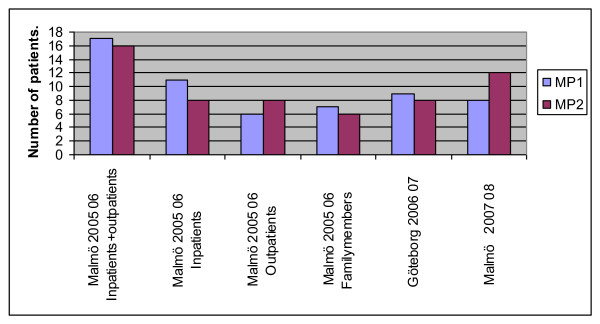
**MP genotype distribution in three seasons with correlation to disease severity in one season**. The samples have been obtained during two winter seasons in Malmö and one in Göteborg, two cities located 250 km apart. The lack of correlation between disease severity and MP genotype is presented in the 2005-2006 MP outbreak in Malmö based on sequences from 33 patients.

In the clinical study group from the community outbreak in Malmö 2005-2006 MP1 was found in 52% (17/33) of the patients. During the winter season 2007-2008 in Malmö 40% (8/20) of the cases had MP1. In Göteborg, studied during the 2006-2007 season 47% (8/17) of the strains were MP1 (Figure 1).

#### Genotype concordance in family contacts

MP was detected in throat swabs from 18 of 22 tested family members in 10 families. Sequencing was successful in 13 of these family cases. In all 7 families, where more than one individual had sequenced samples all strains had the same MP genotype (MP1 in 7 family contacts from 5 families and MP2 in 5 family contacts from 2 families). In one additional family member MP2 was detected but the index case isolate could not be sequenced. The partial adhesion P1 gene sequences within each investigated family were identical.

### Genetic conformity

**Figure 2:**
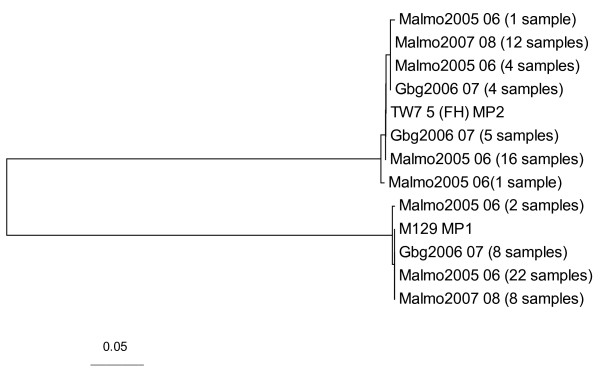
**Phylogenetic analysis of the P1 gene of MP in clinical isolates from three seasons**. Phylogenetic neighbour joining analysis of the P1 gene of MP in clinical isolates from three different winter seasons. Reference sequences prototype genotype 1 (M 129) and type 2 (TW 7-5/FH) were obtained from GenBank (accession number U00089) and Su et al., 1990, respectively.

The P1 sequences were strikingly similar within each genotype and 38 of 40 MP1 isolates were identical in the amplified region whereas two showed a silent mutation. Likewise, the MP2 strains were highly similar. One common point mutation at position 976 codon 325 of the P1 gene was found in 21 of the 43 MP2 isolates leading to a change from valine to isoleucin and in one of these 21 strains an additional mutation leading to a change from threonine to alanine was found. Also in the other MP2 lineage with valine at codon 325 a coupled AC to TG mutation induced a change from threonine to cysteine. These minor point mutations were not confined to any single season or geographical site as shown by the phylogenetic tree (figure 2).

## Discussion

The factors determining disease manifestations and severity in MP infection are only partly understood. Comparing patients with mild and more severe MP respiratory infection, we found that patients in need of hospitalization had higher MP load in throat secretions than outpatients. This difference remained statistically significant after adjustment for age and interval between disease onset and sampling. Both MP genotype 1 and 2 were detected in similar proportions during the different outbreaks. Genotype identity was neither associated with disease severity nor bacterial load.

Higher MP load in hospitalized patients than in outpatients has previously been reported by Dorigmo-Zetsma et al [4] in ten patients; however, no statistical adjustment was done with regard to age and disease duration at sampling which is important as the pathogen load continuously declines with time after onset of disease [3].

Both age and time between disease onset and sampling were adjusted for in our calculations confirming the association between bacterial load and disease severity. This suggests that the pathogenesis of MP is mainly due to direct bacterial factors, a hypothesis that is supported by the finding of a higher risk of severe MP disease in patients for whom adequate antibiotic therapy had been delayed [13].

However, the existence of such an association does not exclude that immune-mediated mechanisms might also be involved in pathogenesis. In patients with high MP loads, elevated amounts of antigen could trigger a more prominent inflammatory response. This may explain why patients with severe MP pneumonia appear to benefit from corticosteroids given in addition to appropriate antibiotic treatment [14,15]. Furthermore, improved resolution of pulmonary lesions in MP-infected mice having received a combination of macrolide antibiotics and corticosteroids compared to those who only received antibiotics have been found, lending further support to this association [16].

Different scoring methods have been used to determine the severity of pneumonia. We used hospital admission as a marker of more severe illness. Several factors apart from the severity of the acute infection may influence the need of hospitalisation, such as age, social situation and underlying diseases. In the statistical analysis, however, we have controlled for the influence of age. None of the included patients had multiple co-existing diseases, and in fact the majority were previously healthy and of young middle age. Only one hospital was involved in the study of clinical severity, making a bias in admission policy unlikely.

CRP levels were significantly higher in admitted patients (188 mg/L vs. 20 mg/L), further supporting our grouping of patients since CRP levels have been shown to predict severity of pneumonia [17-19].

Among our hospitalized MP patients, all 23 who underwent x-ray had pulmonary infiltrates consistent with pneumonia. The true proportion of outpatients with pulmonary infiltrates is unknown since only two of them had symptoms justifying x-ray.

It has been suggested that MP may be a commensal of the respiratory tract mucosa; thus, the identification of MP among outpatients with URTI could reflect colonization rather than invasive infection [20]. However, in our previous study using the same PCR method, we found a low rate of MP carriage (0.4%) among 237 children healthy enough to be in school during a season with high rates of MP cases [3]. The single child diagnosed with MP in that population study was found to have typical symptoms with persistent cough and fever. Our findings in school children are in agreement with those reported by Kumar *et al *who did not detect MP by multiplex PCR in any nasopharyngeal swab from 129 asymptomatic children and adults [21]. Taken together, this indicates that detection of MP DNA in a patient with URTI is likely to signify symptomatic infection.

Several factors could contribute to the differences in MP load observed between patients. Immunosuppression and smoking [22,23] have been associated with higher MP load, but both these factors were rare among our patients. Partial immunity from prior MP infections might also explain the different bacterial loads [24]. In an outbreak in Israel, smoking and low pre-existing MP antibody levels were associated with an increased risk of symptomatic MP infection [23], suggesting that pre-existing antibody levels may affect the disease course.

In contrast to our observed association between bacterial load and clinical severity, no such association was found between genotype and clinical severity. Both MP1 and MP2 had similar distribution with regard to clinical severity.

In Japan, Kenri *et al *[11] found a gradual type shift of the most prevalent MP subtype during the period 1995-2005 from MP2 to MP1 in 127 clinical isolates obtained in three separate areas. Similar shifts of predominant type over time have been reported from Germany; France and Denmark [25]. In our study, covering a much shorter time span both genotypes were detected during each season and in two geographical locations. Although immunological pressure may favour shifts of MP genotype, a co-circulation of MP genotypes appears to be common.

With the very restricted phylogenetic differences found within each subtype, the chosen gene product was too conserved to allow for analysis of transmission chains. Genotype and exact sequence conservation was however observed within each investigated family. To our knowledge genome conformity of MP has not been studied before.

Our study has some limitations. Most patients were recruited from a department of infectious diseases at a university hospital, which may have led to a disproportionate number of cases with more severe MP infection. Most patients in our study were young middle-aged adults. Therefore, our findings may not be applicable to children, who often constitute the majority of MP cases during outbreaks. Few participants in this study had severe underlying diseases or were smokers. This could reflect a selection bias in sampling. However, in the prevalence study from which the participants in this study were recruited [3], MP sampling was performed on a broad range of patients with pulmonary infections and underlying conditions, suggesting that MP infection is not a common pathogen in immunosuppressed subjects. All of the hospitalized patients and 40% of the outpatients had received betalactam antibiotic therapy prior to the MP diagnosis, but since MP lacks a cell wall, on which betalactam antibiotics exercise their effect, it is improbable that this treatment has influenced our results.

Throat swabs were used to obtain samples, and the same technique was used for hospitalized subjects, outpatients and family contacts. The amount of throat secretion absorbed by the swab before dilution in 1 mL of buffered saline could not be determined, making standardization of samples difficult. Housekeeping genes such as beta-globin maybe useful to standardize results from throat swabs. This is important when intracellular agents such as viruses are looked for but may be less useful in the case of MP which is a superficial infection of the mucous membranes.

Since sampling of patients and family members was done in few sites by a limited number of trained staff, sampling problems are unlikely to explain the observed 10-fold differences in bacterial load.

## Conclusions

This study demonstrated a statistically significant association between clinical disease severity and bacterial load in patients with MP infection also after adjustment for age and interval between onset of symptoms to sampling by a logistic regression analysis. This finding suggests that direct bacterial factors are important in MP pathogenesis. MP genotype, however, was not related to disease severity indicating that both genotypes have similar pathogenic potential. During three consecutive winter seasons, two separate highly conserved MP genotypes co-circulated in similar proportions.

## Competing interests

The authors declare that they have no competing interests.

## Authors' contributions

ACN has contributed to the design of the study, the acquisition of samples of the Malmö patients and the analysis and interpretation of data. She was primarily responsible for writing the manuscript. PB has contributed to the design of the study as well as the analyses and interpretation of data. CWO has contributed to the acquisition of samples of the Göteborg patients. AW has contributed to the analyses and interpretation of the phylogenetic data.

KP has contributed to the design of the study, performed the laboratory analysis of the samples and the analyses and interpretation of data. All authors have contributed by revising the manuscript and approved of the version to be published.

## Pre-publication history

The pre-publication history for this paper can be accessed here:

http://www.biomedcentral.com/1471-2334/10/39/prepub
